# *Stenotrophomonas maltophilia* infective endocarditis complicated by two embolic strokes: a rare case report and treatment approach

**DOI:** 10.1128/asmcr.00005-25

**Published:** 2025-07-09

**Authors:** Khaled Qadabashi, Hadi Itani, Moied Al Sakan, Mike Ghabally, George F. Araj

**Affiliations:** 1 Department of Internal Medicine, American University of Beirut Medical Center 66984, Beirut, Lebanon; 2 Department of Internal Medicine, Northwell Health, Staten Island University Hospital 7601 , New York, New York, USA; 3 Division of Cardiology, Department of Internal Medicine, American University of Beirut Medical Center 66984, Beirut, Lebanon; 4 Division of Cardiology, Department of Internal Medicine, Aleppo University Hospital 675385, Aleppo, Syria; 5 Department of Pathology and Laboratory Medicine, American University of Beirut Medical Center 66984, Beirut, Lebanon; Pattern Bioscience, Austin, Texas, USA

**Keywords:** *Stenotrophomonas maltophilia*, endocarditis, stroke, infection

## Abstract

**Background:**

*Stenotrophomonas maltophilia* is a rising opportunistic organism among hospitalized patients, leading to outbreak infections. It is known to be a nosocomial pathogen resistant to multiple antibiotics and may be transmitted via contaminated hospital equipment. Its infection can lead to cardiac complications, although very rare.

**Case Summary:**

We present a case of a 63-year-old patient who presented with insidious symptoms of fever, chills, and chest discomfort and was admitted for the management of prosthetic valve infective endocarditis caused by *S. maltophilia* evident by positive blood cultures and visualized vegetations on the mitral valve by echocardiography. The hospital stay was complicated by two embolic strokes. The patient underwent successful surgical replacement of the prosthetic mitral valve. However, given the patient’s significant disability and morbidity, he required assistance in performing the activities of daily living in addition to placement of a gastrostomy tube due to the high risk of aspiration.

**Conclusion:**

*S. maltophilia* infective endocarditis is a rare but serious infection that warrants immediate attention with prompt initiation of intravenous antibiotics. In addition, and when indicated, surgical intervention can lead to successful management and minimize the risk of complications, morbidity, and mortality.

## INTRODUCTION

*Stenotrophomonas maltophilia* is a gram-negative, non-fermentative, aerobic bacillus that is present in the natural environment (1, 2). It is a nosocomial pathogen that is resistant to several antibiotics through intrinsic mechanisms such as metallo-β-lactamases (L1 and L2) that hydrolyze β-lactams, efflux pumps (SmeDEF and SmeVWX) that expel antibiotics, mutations in target sites (gyrA and parC) for fluoroquinolones, and altered porin channels (3). Thus, antibiotic therapy for this organism is difficult (4).

Despite its low virulence, *S. maltophilia* is emerging as an opportunistic pathogen that leads to outbreaks of serious infections (e.g., pneumonia and bacteremia) among hospitalized patients, particularly in the immunocompromised such as transplant patients and with malignancy receiving treatment (1, 2, 5). The mode of transmission of this organism may be via contaminated hospital equipment or from environmental water sources such as tap water or faucets (4, 6).

*S. maltophilia* can lead to severe cardiac complications. One of these complications is infective endocarditis (4, 7–9). Infective endocarditis secondary to *S. maltophilia* infection is considered to be rare, as around 40 cases of *S. maltophilia* infective endocarditis have been reported in the medical literature, and it carries elevated morbidity and mortality rates. This organism tends to favor prosthetic valves more than the native ones. Risk factors for acquiring it include previous cardiac surgery, infected intravascular devices, dental treatment, and intravenous illicit drug use (4, 10). Previous valve replacement is one of the most significant factors for *S. maltophilia* endocarditis, and it is responsible for around 40%–60% of cases (4, 8).

## CASE PRESENTATION

Our patient is a 63-year-old man who presented to the emergency department at the American University of Beirut Medical Center (AUBMC) with a 2 month history of fever, chills, and intermittent retrosternal chest discomfort. He denied any dyspnea, palpitations, weight loss, orthopnea, paroxysmal nocturnal dyspnea, abdominal bloating, or lower extremities edema. His past medical history was pertinent for coronary artery disease (mild non-obstructive atherosclerosis confirmed by coronary angiography before surgery), heart failure with preserved ejection fraction, and mitral and aortic valve replacement with bio-prosthetic valves 1 year ago secondary to degenerative mitral and aortic valve regurgitation. He had no recent history of dental treatment. Social history was pertinent for occasional cigarette smoking. Physical examination upon admission was within normal limits. His electrocardiogram was normal. Chest X-ray demonstrated a small left pleural effusion, along with residual epicardial pacing wires.

**Fig 1:**
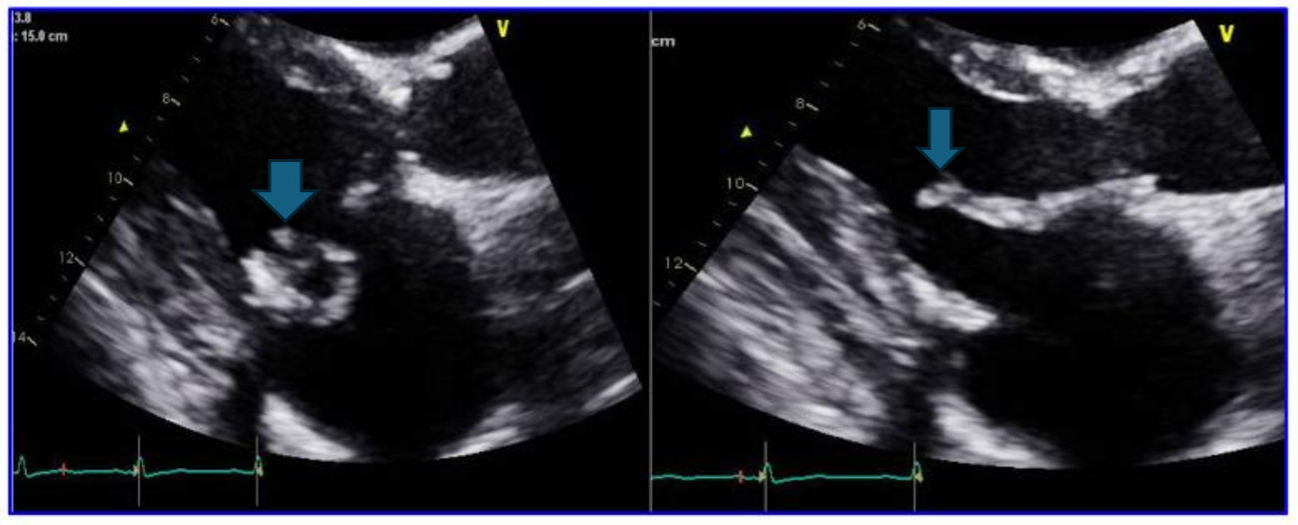
Transthoracic echocardiography pictures in the parasternal long axis view (PLAX) depicting a filamentous structure measuring 1.5 cm noted on both mitral valve leaflets, as indicated by blue arrows.

Figure 1 shows the transthoracic echocardiogram revealing a bio-prosthetic mitral valve with evidence of vegetation and mild regurgitation, a bio-prosthetic aortic valve with normal parameters and mild tricuspid valve regurgitation with normal pulmonary arterial pressure and an ejection fraction of 60%–64%.

During hospitalization, one blood culture was taken every 12 hours, with the first consecutive five blood culture bottles signaled positive. The first positive culture was detected after 55 hours of incubation in the Virtuo Blood Culture automated instrument (BioMérieux, France). Upon subculture, there was bacterial growth on the blood agar, chocolate agar, and MacConkey agar plates, which was determined to be *S. maltophilia* by a matrix-assisted laser desorption/ionization time of flight (MALDI-TOF) system (Bruker Daltonik, GmbH, Bremen, Germany) with a score of 2.20 (green flag). Antimicrobial susceptibility of the isolate was carried out using the disk diffusion method and interpreted according to Clinical and Laboratory Standards Institute (CLSI, 2024) guidelines (11). It revealed susceptibility to levofloxacin, trimethoprim/sulfamethoxazole (TMP/SMX), and minocycline. Consequently, levofloxacin 500 mg intravenous drip twice a day and TMP/SMX 800–160 mg two tablets orally three times daily were started to achieve blood culture sterility prior to any surgical intervention. The subsequent blood cultures, taken 24 hours after initiating treatment, remained negative for *S. maltophilia*.

**Fig 2:**
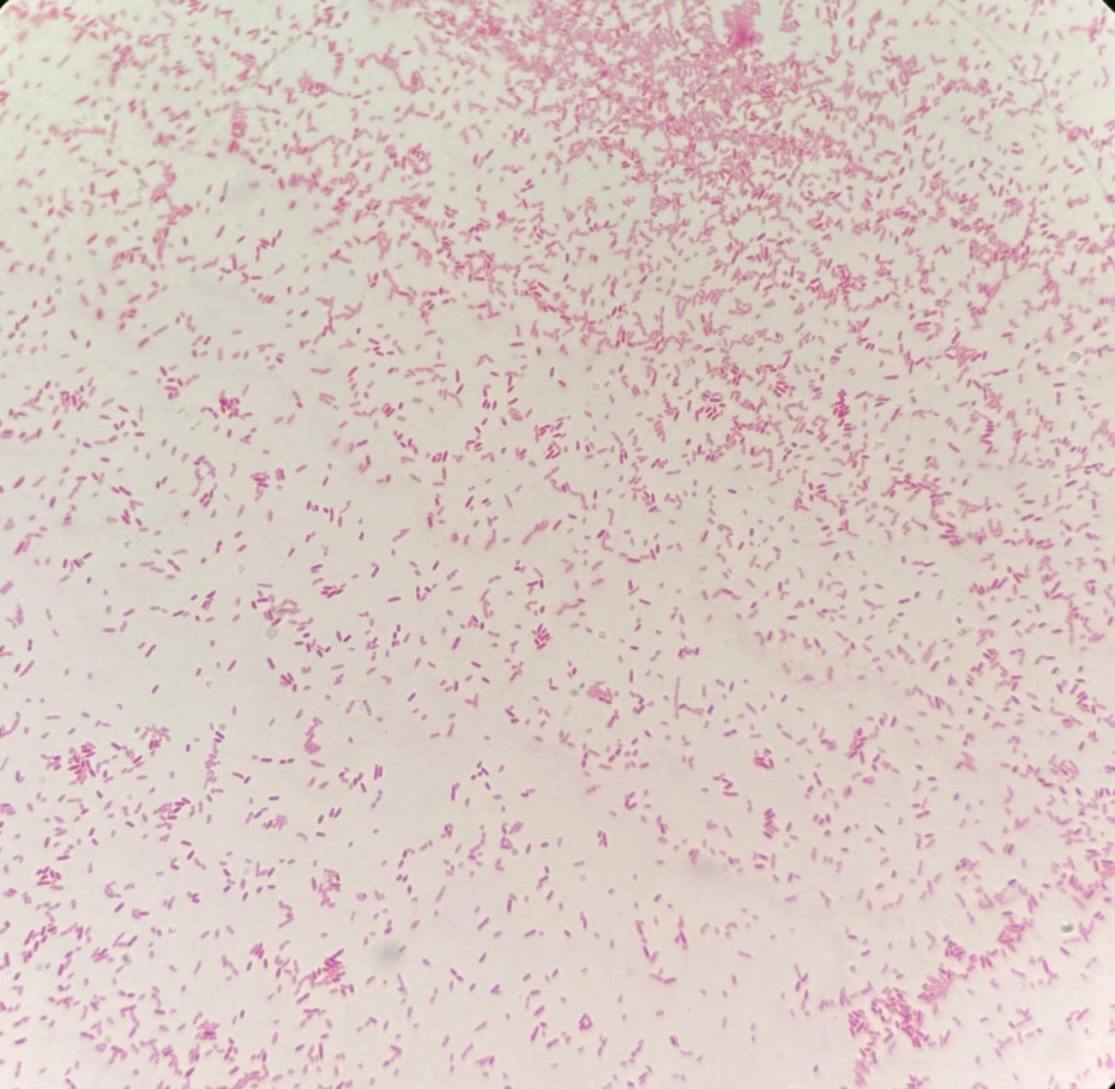
Gram-negative bacilli appearing as pink-stained rods, arranged singly and in pairs.

Figure 2 displays the characteristic morphology of of *S. maltophilia* on gram stain.

**Fig 3:**
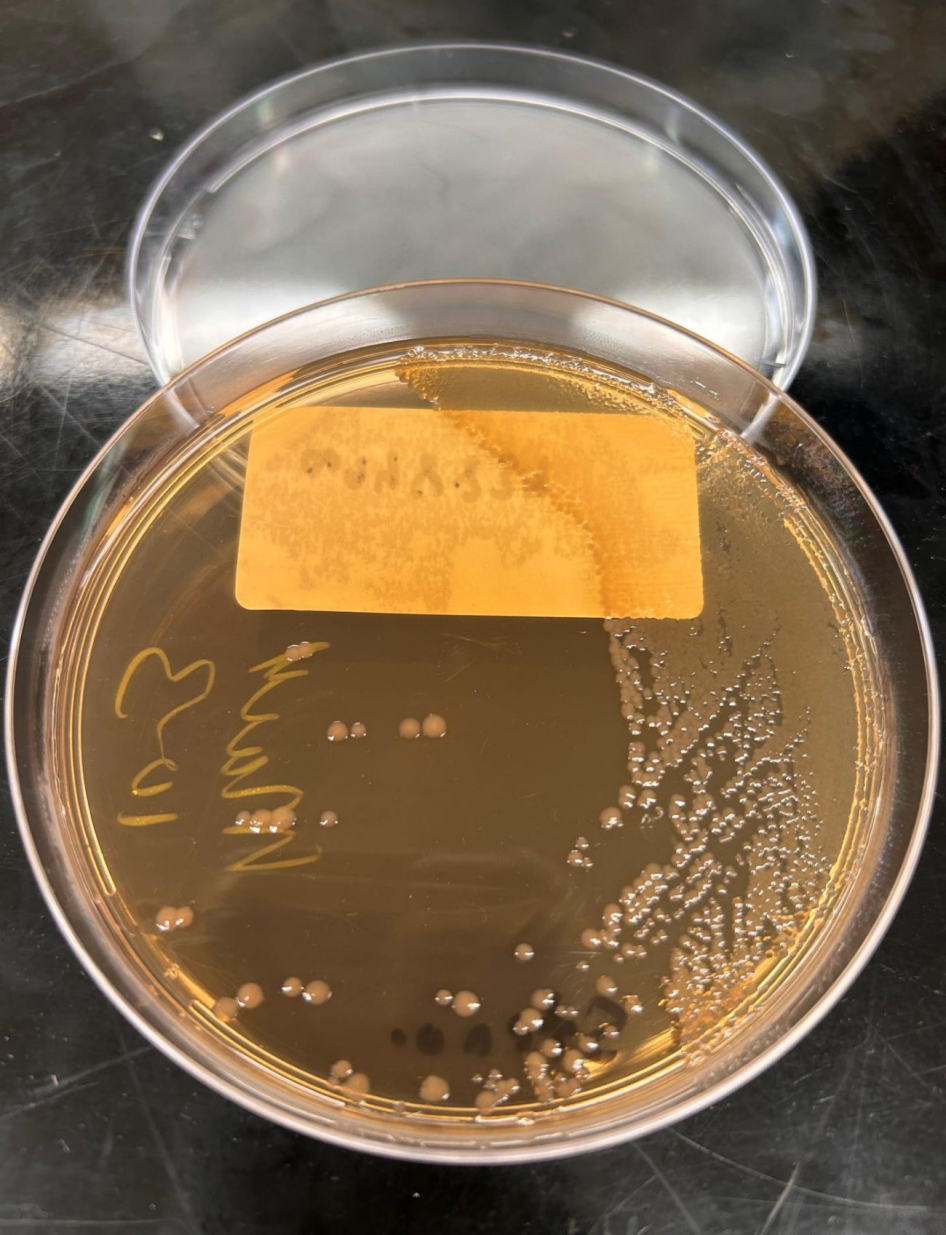
*S. maltophilia* colonies on MacConkey agar after 55 hours of incubation.

Figure 3 demonstrates the colony morphology of *S. maltophilia* on MacConkey agar following 55 hours of incubation.

Ten days later, the patient developed sudden onset global aphasia with right-sided distal weakness, mainly in the right upper extremity, and the National Institutes of Health Stroke Scale was 6. An urgent magnetic resonance imaging (MRI) of the brain without gadolinium was done, and it showed a hyper-acute stroke in the left post-central gyrus along with a right corona radiata subacute infarct. Therefore, he was commenced on aspirin 100 mg oral daily and clopidogrel 75 mg oral daily while continuing the antibiotics.

**Fig 4:**
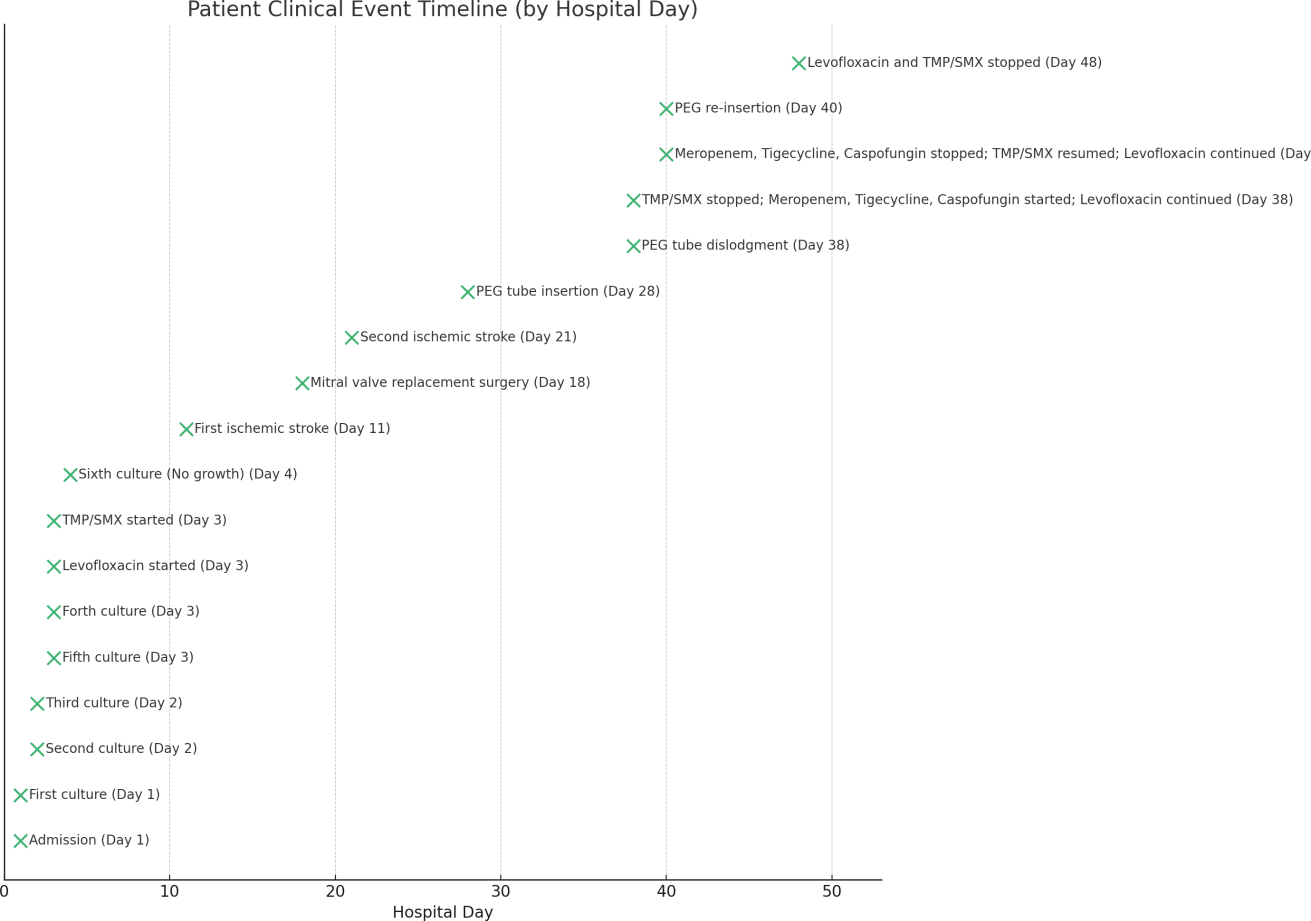
Timeline of the patient’s hospital stay, summarizing key events, culture results, and antibiotic therapy.

One week later, mitral valve replacement surgery was done, and no microbiological analysis was performed on the vegetations obtained during surgery. Three days after the surgery, he developed new-onset global aphasia and right hemiplegia. Urgent MRI revealed embolic right and left strokes with left internal carotid artery filling defect suggestive of embolus. Accordingly, due to the high risk of hemorrhagic transformation, he was switched to a single antiplatelet agent (aspirin 100 mg oral daily). Given the patient’s functional status decline and the prolonged time needed for neurological recovery, including swallowing, a percutaneous endoscopic gastrostomy (PEG) tube was inserted for long-term feeding and medication administration. After ten days, his condition was complicated by the dislodgment of the PEG tube, leading to hemodynamic instability. Consequently, TMP/SMX was stopped, and meropenem 2 g intravenous drip twice daily, tigecycline 100 mg intravenous drip twice daily, and caspofungin 50 mg intravenous daily were added while keeping levofloxacin. This choice of antibiotic coverage, despite negative blood culture, was empirically used to cover for possible multidrug-resistant organisms, given the patient’s prolonged hospitalization, and treat possible concealed abdominal collection and to cover any possible fungal translocation from the side of the PEG tube opening in the stomach. Two days later, his condition improved with no evidence of fungal infection based on the reassuring abdominal computed tomography imaging and negative blood cultures. Therefore, the patient was diagnosed with chemical peritonitis. Subsequently, meropenem, tigecycline, and caspofungin were stopped, and TMP/SMX was resumed in addition to levofloxacin, with successful re-insertion of the PEG tube. One week later, the patient’s condition was stable, and all subsequent blood cultures remained negative. In view of the prolonged antibiotic course that the patient received throughout the hospitalization, a decision was made to cease antibiotherapy. Overall, the patient received a total of 44 days of levofloxacin and 42 days of TMP/SMX during this hospitalization before discharge. After 2 years of follow-up, the patient continues to do well with no reported complications. Figure 4 presents the patient’s clinical course, highlighting the clinical events that occurred during his stay at the hospital.

## DISCUSSION

Infective endocarditis from *S. maltophilia*, although rare, carries the critical risk of morbidity. Complications from *S. maltophilia* endocarditis include cerebrovascular disease, congestive heart failure, septic embolism, organic cerebral, lung, and cardiac abscesses, as well as aortic dissection (4, 10). Subhani et al. summarized the reported cases of infective endocarditis secondary to *S*. *maltophilia* throughout the medical literature and noted that the complication rates occurred in more than half of the patients at 52%, whereas the mortality rate was 33% (11).

Generally, treatment consists of antibiotic therapy and removal of the infected indwelling foreign material in order to eradicate the infection and avoid its recurrence (4, 10). In this case, the patient was successfully managed with a combination of medical and surgical interventions.

TMP/SMX is widely accepted as the first-line treatment for *S. maltophilia* infections due to high *in vitro* susceptibility rates, reaching up to 93.8% in various studies (10, 12, 13). However, since TMP/SMX is a bacteriostatic drug, its effectiveness may be limited in severe cases such as bacteremia and endocarditis, where bactericidal activity is crucial (12). Regarding the use of single or combined therapy, Zelenitsky et al. reported that antibiotic combinations were significantly more effective in reducing bacterial loads of *S. maltophilia* than monotherapy (7, 14). A systematic review by Khan et al. revealed that a combination of TMP/SMX and levofloxacin reduced mortality compared to other regimens (15). Additionally, minocycline has demonstrated a high *in vitro* susceptibility against *S. maltophilia,* including TMP/SMX-resistant strains. High-dose minocycline has demonstrated clinical success comparable to TMP/SMX in the management of bacteremia, with associated lower mortality too (16). However, the clinical evidence remains limited and contradictory, and the IDSA suggests either a combination therapy of at least two active agents (cefiderocol, minocycline, TMP/SMX, or levofloxacin) or by using the combination of ceftazidime-avibactam and aztreonam (17). Nevertheless, limited data remain comparing monotherapy vs combination therapy in the management of *S. maltophilia* infections, including infective endocarditis. Therefore, the treatment should be individualized based on susceptibility testing and clinical judgment. It should be noted that the use of levofloxacin in combination therapy is declining due to the rising resistance, whereas ciprofloxacin is not recommended due to poor susceptibility and lack of clinical data (18). The difficulty in treating *S. maltophilia* persists, as even bactericidal drugs like ceftazidime have been reported to be ineffective due to the intrinsic resistance mechanisms of *S. maltophilia*, which produces inducible chromosomal metallo-β-lactamases (L1 and L2) that confer resistance to all β-lactam antibiotics (including carbapenems), leaving only two options: cefiderocol and the combination of ceftazidime-avibactam and aztreonam (19, 20). Moreover, limited data have demonstrated successful treatment of *S. maltophilia* bacteremia and infective endocarditis following the administration of avibactam-aztreonam (16).

Given the rarity of endocarditis infection due to this pathogen, limited data are available regarding the optimal antimicrobial therapy and timing of surgical intervention. In our case, the recovered *S. maltophilia* showed susceptibility to levofloxacin and TMP/SMX. Thus, the option was to use the combination of these as therapeutic drugs, aligning with what was reported earlier of these being the preferred treatment in severe cases (21).

The search for new treatments for *S. maltophilia* is ongoing, and recently, cefiderocol has been recognized as a potential therapeutic option (22). Cefiderocol is a catechol-substituted siderophore cephalosporin, similar in its structure to cefepime and ceftazidime (22). In addition, a clinical case report has demonstrated successful treatment outcomes using cefiderocol for serious *S. maltophilia* infection (23). Additionally, experimental studies have shown that cefiderocol is highly active in treating *S. maltophilia* pneumonia, supporting further clinical investigations into its efficacy (24).

A preliminary study at the clinical microbiology laboratory of AUBMC in Lebanon demonstrated uniform susceptibility of all *S. maltophilia* isolates tested *in vitro* against cefiderocol, including our patient isolate. Antimicrobial susceptibility testing was performed using the disk diffusion method and showed a wide inhibition zone diameter that ranged from 28 to 33 mm, whereby the susceptibility interpretation zone based on CLSI 2024 defined the susceptibility breakpoint as ≥15 mm (25).

Our patient had multiple risk factors, including bioprosthetic aortic and mitral valves, that increased his susceptibility to infective endocarditis.

A limitation in this case is the oversight of requesting microbiological testing on the intraoperative specimens obtained during valve replacement. This represents a missed diagnostic culture opportunity for recovering a pathogen in endocarditis.

### Conclusion

*S. maltophilia* infective endocarditis is a rare but serious infection that should be detected and managed early on in order to improve the patient’s outcomes. It tends to occur more commonly in patients with structural heart defects. Management consists of both antimicrobial therapy and surgical intervention when needed to eradicate the infected area. Serious complications (e.g., stroke) can occur as a consequence of such an infection and could carry a detrimental effect on the patient’s quality of life, together with a high rate of morbidity and mortality.
